# Encapsulation Systems for Antimicrobial Food Packaging Components: An Update

**DOI:** 10.3390/molecules25051134

**Published:** 2020-03-03

**Authors:** Raquel Becerril, Cristina Nerín, Filomena Silva

**Affiliations:** 1 I3A–Aragón Institute of Engineering Research, University of Zaragoza, Calle María de Luna 3, 50018 Zaragoza, Spain; raquel@unizar.com (R.B.); cnerin@unizar.es (C.N.); 2 ARAID–Agencia Aragonesa para la Investigación y el Desarollo, Av. de Ranillas 1-D, planta 2ª, oficina B, 50018 Zaragoza, Spain; 3 Faculty of Veterinary Medicine, University of Zaragoza, Calle de Miguel Servet 177, 50013 Zaragoza, Spain

**Keywords:** active packaging, antimicrobials, encapsulation, electrospinning, nanocarriers, essential oils, metal nanoparticles, emulsions, natural compounds

## Abstract

Antimicrobial active packaging has emerged as an effective technology to reduce microbial growth in food products increasing both their shelf-life and microbial safety for the consumer while maintaining their quality and sensorial properties. In the last years, a great effort has been made to develop more efficient, long-lasting and eco-friendly antimicrobial materials by improving the performance of the incorporated antimicrobial substances. With this purpose, more effective antimicrobial compounds of natural origin such as bacteriocins, bacteriophages and essential oils have been preferred over synthetic ones and new encapsulation strategies such as emulsions, core-shell nanofibres, cyclodextrins and liposomes among others, have been applied in order to protect these antimicrobials from degradation or volatilization while trying to enable a more controlled release and sustained antimicrobial action. On that account, this article provides an overview of the types of antimicrobials agents used and the most recent trends on the strategies used to encapsulate the antimicrobial agents for their stable inclusion in the packaging materials. Moreover, a thorough discussion regarding the benefits of each encapsulation technology as well as their application in food products is presented.

## 1. Antimicrobial Food Packaging

In Europe, the food sector is a major sector that generates more than 750,000,000,000 euros each year [1], representing 4.4% of the Gross Domestic Product to the European Economy [2]. According to the latest data provided by FAO [3], about one third of all food produced for human consumption is wasted each year, which corresponds to 1.3 Gtons of food; a global tendency that is expected to grow in the future [4].

Given the economic impact of the food industry in our society, microbial contamination of foods can result in significant losses for the food industry due to food spoilage. Furthermore, the consumption of microbial contaminated foods can lead to serious public health threats such as foodborne diseases and outbreaks. Microbial food spoilage is mainly caused by non-pathogenic spoilage microorganisms that are responsible for alterations on the nutritional and sensory characteristics of food products, such as oxidation, generation of off-flavours and off-odours as well as undesirable changes in texture and colour [5]. On the other hand, foodborne disease is caused by pathogenic microorganisms that are responsible, each year, for 600,000,000 cases of illness, with almost 420,000 deaths and 27,000,000 Years of Life Lost (YLL), according to World Health Organization (WHO) [6].

The first attempt of the food industry to fight microbial contamination was based on the direct addition of antimicrobials (e.g., food preservatives) to food products. This strategy proved to be of limited action due to the rapid diffusion of the antimicrobial substance from the surface to the mass of the product [7], with concomitant loss of efficacy, so the food industry had to search for new and innovative ways to introduce antimicrobials in food products. Given that 99.8% of all food and beverages have to be encased in some sort of packaging during their existence, the next logical step was to include these antimicrobial substances in the food packaging material, giving rise to antimicrobial food packaging technology. A clear advantage of this option would be that the packaged food would be protected without having edible preservatives added directly in its composition. Antimicrobial packaging has the main goal of reducing, retard or even inhibiting microbial growth by interacting with the packaged food (direct contact) or the package headspace (indirect contact) [5]. By controlling microbial flora, antimicrobial packaging ensures microbial food safety, while maintaining food’s quality and sensorial properties and increasing products’ shelf-life [8]. Nowadays, antimicrobial packaging can come in several forms such as sachets or pads containing volatile antimicrobials, polymer films with direct incorporation of antimicrobial substances (extrusion, casting) and coating, adsorption or grafting of antimicrobials onto the surface of the polymer [7]. It is quite obvious that antimicrobials have to reach the cells to inhibit their growth or to kill them. This fact implies that the antimicrobial agents will have to be in contact with the food, either in vapour phase or by direct contact between the active packaging and the food [8]. There is a wide and ever-growing list of antimicrobial agents that have been or are currently being for the development of antimicrobial food packaging. Although the list is vast, not all antimicrobials are suitable for every application, as the choice of the antimicrobial to be used depends on several factors. The primary factor is the antimicrobial activity against the target microorganisms, including specific activity and resistance development, and the regulatory status of its use in foods [9]. Furthermore, one has to take into account whether controlled release approaches are necessary or not, given the chemical nature of the food, its storage and distribution conditions as well as the physical-chemical characteristics of the packaging material where the antimicrobial is going to be included [9].

### 1.1. Antimicrobial Substances Used in Food Packaging

The list of antimicrobial substances used for the development of antimicrobial food packaging is quite vast and is continuously evolving as a result of changing consumer trends and legislation. These substances include chemicals such as organic acids, triclosan, antibiotics, chlorine dioxide, nitrites and ammonium salts that are slowly being replaced by “greener”, more natural alternatives such as bacteriocins, enzymes, phages, biopolymers, natural extracts and compounds, essential oils and their components and metal nanoparticles (Table 1).

#### 1.1.1. Organic Acids and Their Salts

Some organic acids, such as propionic acid, benzoic acids, sorbic acid, lactic acid, and acetic acid, and their salts are synthetic antimicrobial agents that have strong antimicrobial activity and can be used for the development of antimicrobial packaging materials (Table 1). These colourless and tasteless substances are considered to be GRAS by the FDA [36] and have been used as preservatives in the cosmetics, pharmaceutical, and food industries for many decades. They have a very broad antimicrobial range, being active against yeasts, moulds, Gram-positive and Gram-negative bacteria [13] with distinct antimicrobial spectra depending on the acid or salt. For instance, salts of lactic acid, such as sodium lactate and potassium lactate, exert greater inhibitory effects against Gram-positive bacteria than against gram-negative bacteria and offer antifungal activity against certain *Aspergillus* species [37]. Potassium sorbate inhibits the germination of bacterial spores [38]. Organic acids such as lactic acid, sodium benzoate, citric acid or potassium sorbate have been included in packaging materials or composites giving rise to antimicrobial food packaging materials active against Gram-positive and Gram-negative bacteria (Table 1)

Several organic acids, their salts or anhydrides are listed as food preservatives in the EU database that informs about the food additives approved for use in food in the EU and is based on the EU list of food additives contained in the Annex II of Regulation (EC) No 1333/2008 [39] (Table 2). In addition to being classified as preservatives due to their antimicrobial action, these compounds can also serve other functions as food additives such as acidifiers, acidity regulators, stabilizers, antioxidants, vitamins, flavour enhancers, baking and flour treatment agents, emulsifying salts and sequestrants (for a more detailed review please see [40]).

#### 1.1.2. Bacteriocins

Bacteriocins are natural antimicrobial peptides with positively charged compounds and hydrophobic moieties produced by Archaea, Gram-positive and Gram-negative bacteria. These positively charged compounds can interact electrostatically with the negative charges of the phosphate groups on the microbial cell membranes, resulting in the generation of pores in the membrane and subsequent cell death [38].

Although Gram-negative bacteria also produce bacteriocins like colicins, tailocins, alveicins and cloacins [41], the broad spectrum bacteriocins from Gram-positive bacteria have a more suitable use for food applications than the ones from Gram-negative bacteria, since the bacteriocin producing strains can be directly added to the food matrix and no exhaustive purification process is required, given that these preparations would not contain lipopolysaccharides (LPS) or other endotoxins that could cause health issues when ingested [41]. Although there is a vast list of bacteriocins, among the most well-known, we can find nisin, pediocin PA-1, sakacin A, enterocin AS-48, lacticin 3147A and bacteriocin 7293 (Table 3).

Over the last decades, bacteriocins have been used for food preservation because of their GRAS status recognition by the FDA and their lack of activity and toxicity to consumers [42]. Furthermore, after ingestion, they are inactivated by digestive tract proteases and do not influence the consumer’s gut microbiota [42]. Regarding their antimicrobial effectiveness, these compounds are active over a wide range of temperature and pH and have a relatively broad spectrum of antimicrobial activity against foodborne pathogens and spoilage bacteria (Table 3), especially against Gram-positive bacteria such as *Listeria*, *Bacillus* and *Clostridium* species as well as lactic acid bacteria.

There are two main methods of using bacteriocins into food packaging applications (Table 1): (i) in situ, by incorporating bacteriocin-producing bacteria [21], or (ii) ex situ with the addition of purified or semi-purified bacteriocin or bacteriocin-like substances [15,16,20]. Taking into consideration that bacteriocins are more effective against Gram-positive bacteria and possess a narrow antimicrobial spectrum, they are used in food packaging in combination with other antimicrobials or preservation techniques. Over the last five years, the use of enterocin AS-48 [47], bacteriocins-like substances [48] or bacteriocin-producing strains [49] with modified atmosphere packaging or high hydrostatic pressure has yielded improved results in the packaging of chilled food products. Furthermore, bacteriocin combination with other antimicrobials such as thymol [47,50], carvacrol [50], EDTA [51] or chitosan [17,52] have also improved their antibacterial action in food products such as meat and fish.

According to the EU legislation, so far, nisin is the only bacteriocin approved as a food additive (E 234) [39]. The European Food Safety Authority (EFSA) also granted the Qualified Presumption of Safety (QPS) status to most of the lactic acid bacteria genera, such as *Lactococcus, Lactobacillus, Leuconostoc, Pediococcus,* and some *Streptococcus* [53]. Nevertheless, species of the genus *Enterococcus* and some *Streptococcus* are pathogenic, thus, they have not been proposed for QPS status [53]. The QPS approach was developed by the EFSA Scientific Committee to provide a harmonised generic pre-evaluation to support safety risk assessments of biological agents intentionally introduced into the food and feed chain, in support of the concerned scientific Panels and Units in the frame of market authorisations [53].

#### 1.1.3. Enzymes

Enzymes such as lysozyme, glucose oxidase, lactoferrin or the lactoperoxidase system can be used as effective antimicrobials in food packaging through their incorporation by chemical binding or grafting or physical entrapment in packaging materials (Table 3). Lysozyme is one of the most widely used enzymes as a food preservative due to its proven antimicrobial activities against bacteria, fungi, protozoans, and viruses [37]. However, this enzyme is more effective against Gram-positive bacteria due to its ability to break down the glycosidic bonds of peptidoglycan in the cell wall of these bacteria [54]. That is the reason why lysozyme is sometimes used in packaging in combination with other enzymes or compounds, such as lactoferrin or EDTA [23,55]. Lactoferrin is used together with lysozyme to improve its antimicrobial activity against Gram-negative bacteria. Lactoferrin, a whey protein that binds ferric ions, exerts its antimicrobial activity by depriving microbial cells of iron and by altering the permeability of Gram-negative bacteria due to its interaction with LPS components [23].

The glucose oxidase (GO) enzyme, a flavoprotein purified from different types of fungi, especially form *A. niger* and *Penicillium* species, exerts its antimicrobial activity by catalysing the formation of hydrogen peroxide and gluconic acid through the oxidation of β-d-glucose [25]. So far, this enzyme has proven an effective antimicrobial effect against pathogenic foodborne bacteria such as *Salmonella infantis, Staphylococcus aureus, Clostridium perfringens, Bacillus cereus, Campylobacter jejuni*, and *Listeria monocytogens* [25].

Another commonly used enzyme as natural antimicrobial is lactoperoxidase. Lactoperoxidase catalyses the oxidation of thiocyanate ion (SCN^−^) which generates oxidised products such as hypothiocyanite (OSCN^−^) and hypothiocyanous acid (HOSCN). These oxidised products act as antimicrobial agents by causing the irreversible oxidation of sulfhydryl (SH) groups present in microbial enzymes and other proteins, resulting in the loss of activity by these biomolecules and eventually cell death [26].

According to EFSA regulation, food enzymes are categorized as food improvement agents. The Regulation on food enzymes, Regulation (EC) No 1332/2008 harmonises the rules for food enzymes in the European Union (EU) [56]. According to that regulation, all food enzymes currently on the EU market as well as new enzymes have to be submitted to safety evaluation by the European Food Safety Authority (EFSA) and subsequently approved by the European Commission by means of a Union list. Currently, there is no Union list of authorised food enzymes, but there are some food enzymes approved as food additives. So far, from the four enzymes described only lysozyme is accepted by EFSA as a food additive (E1105) under Directive 95/2/EC on food additives [39].

#### 1.1.4. Biopolymers

Two of the most known biopolymers with intrinsic antimicrobial activity are chitosan and pectin. Chitosan is obtained by the deacetylation of chitin, forming a linear structure of randomly acetylated and deacetylated units. Chitosan has been reported as an antimicrobial agent against a wide variety of bacteria, moulds and yeasts [57]. This antimicrobial action is due to the interaction of the positively charged amino groups on chitosan, at a pH below 6, with the anionic cell membranes, which leads to an increased cell permeability and, ultimately, to intracellular components leakage and cell death [9]. Due to its biodegradability and bio-based origin, chitosan can be used to produce environmentally friendly food packaging films either by extrusion [27] or press moulding that will not dissolve in water, unlike other biopolymers. Besides being used alone for the formation of packaging films or edible coatings, chitosan has also been used as a coating for plastic films or other materials [58,59]. However, nowadays, the most promising strategy for the development of chitosan-based antimicrobial films is the one based on chitosan combination with other natural antimicrobials such as bacteriocins [17], essential oils and their components [60,61,62], among others.

In a similar way to chitosan, a polycation, also other bio-based polymers can be used for the development of antimicrobial food packaging as some studies show that polyelectrolytes (polycations and polyanions) have antimicrobial properties [28]. Taking this into consideration, multilayer films composed of alginate, a natural anionic polymer, and cationic hydroxyethyl cellulose, a water soluble film-forming polymer, have been developed as a new packaging material with intrinsic antimicrobial properties [28]. In vitro testing has shown that, depending on the formulation and design used, these films are active against both Gram-positive (*S. aureus*) and Gram-negative bacteria (*E. coli*).

#### 1.1.5. Natural Extracts and Compounds

The increased awareness of consumers regarding synthetic-based antimicrobials and the knowledge of their serious adverse effects on human health has discourage food scientists and consumers to use them and search for novel natural alternatives [54]. In this regard, plants, herbs and spices are being considered as the most important and rich natural source of antimicrobial substances like saponins, tannins, alkaloids, alkenyl phenols, glycoalkaloids, flavonoids, sesquiterpenes, lactones, terpenoids and phorbol esters [63]. Besides having antioxidant and/or antimicrobial properties, these substances can also enhance the organoleptic acceptability of food products [63]. Additionally, the new circular economy strategy for plastic reduction and the search for biodegradable, bio-based packaging materials also encourages the incorporation of natural substances in packaging materials for a “greener”, plastic-free and more sustainable food industry.

Regarding the antimicrobial mechanism of action of natural extracts and phytochemicals it is thought that these natural antimicrobials have a multi-target action on microbial cells being able to disrupt membrane function and structure, interrupt DNA/RNA synthesis/function, interfere with intermediary metabolism, induce coagulation of cytoplasmic constituents and interfere with cell-to-cell communication [64,65,66]. This wide action on the microbial cell subsequently results in a broad spectrum of antimicrobial activity of these compounds and also to a decreased risk in the arise of microbial resistance mechanisms.

Bearing in mind their potential application in food packaging, it is important to point out that most plant-derived extracts are generally recognized as safe (GRAS) and Qualified Presumption of Safety (QPS) status in the USA and EU [63]. Taking all this into consideration, over recent years, many phytochemicals have been used for the development of antimicrobial food packaging materials, mainly with antibacterial action, and tested for their in vitro and in vivo (food product) efficiency in improving microbial safety and shelf-life. Table 4 summarizes some of the most recently used natural extracts and other phytochemicals, such as green tea extract, stilbenes (resveratrol and pinosylvin), kombucha tea extract, *Ginkgo biloba*, olive leaf, grapefruit seed, propolis and several other plant extracts, lignign, gallic acid, with the exception of essential oils and their components, on the development of novel potential antimicrobial food packaging.

#### 1.1.6. Essential Oils and Their Components

Essential oils (EOs) are mixtures of volatile compounds generally obtained from spices and herbs with several biological properties, including antimicrobial activity. According to the International Organization for Standardization (ISO) (ISO DIS9235.2), an essential oil is “a product made by distillation with either water or steam or by mechanical processing of citrus rinds or by dry distillation of natural materials,” meaning that an extract can only be named essential oil if it is obtained by either steam or hydrodistillation [81]. EOs can be obtained from distinct plant materials such as flowers, buds, leaves, stem, bark and seeds [82]. EOs are a complex mixture of compounds such as terpenoids, esters, aldehydes, ketones, acids, and alcohols, where major constituents can compose up to 85% of the oil composition, and the other 15% is composed by minor components and trace elements [82]. This composition depends on plant cultivar, development stage, geographical origin, collection season, plant’s age and cultivation conditions [83]. However, ISO also defines, for some essential oils, the major components and their percentage range, as a standardization method [81].

EOs’ antimicrobial activity is mainly a consequence of their hydrophobicity that enables them to partition into the lipid layer of cell membranes and mitochondria, increasing their permeability, leading to the ion and small molecule leakage and, to a greater extent, cell lysis and death [81]. This also disturbs the cytoplasmic membrane by disrupting proton motive force, electron flow, active transport and efflux [84]. Furthermore, this lipophilic character, makes that EOs accumulate in lipid bilayers and also disturb protein-lipid interactions [84]. Additionally, there is a synergism between EO major and minor components, meaning that the effect of the EO is higher than the sum of the effects of each EO component [82,85].

Considering their regulatory status in the EU for approved use in food packaging, it is important to point out that EOs contain flavouring substances that are approved to be used as flavourings by the European Food Safety Authority [39]. This Regulation prohibits the addition of certain natural undesirable substances and lays down maximum levels for certain substances. In the United States, EOs and their components are also registered as flavouring substances by the Food and Drug Administration and have GRAS status [86].

Regarding natural antimicrobial packaging, EOs have been one of the preferred antimicrobial classes to be included in packaging materials due, in one part, to their approved use as food additives, but also to their major advantage when compared to other phytochemicals and natural extracts, their volatility, meaning that no direct contact between the packaging material and the food product is required for EOs to exert their antimicrobial activity [87]. To date, many EO-containing packaging materials have been developed at laboratory scale and even as commercial solutions [8] incorporating cinnamon, oregano, lemongrass, ginger, thyme, chamomile, tea tree, among others, as well as some of their bioactive major components such as thymol, carvacrol, geraniol, terpilenol and eugenol [for a more detailed review, see [82,88]]. The latest developments on the use of essential oils and their constituents for antimicrobial packaging materials as well as the encapsulation strategies used to stabilize these oils in the packaging material, at research level, and their use in food preservation are summarized in Table 5.

#### 1.1.7. Metal Nanoparticles

Metal nanoparticles (NPs) have been widely used as antimicrobial agents due to their high thermal stability, longevity, and their broad spectrum of antifungal and antibacterial activities [104]. Among the most used nanoparticles are silver, copper, gold, titanium dioxide (TiO_2_), zinc oxide (ZnO) and magnesium oxide (MgO) [37], with silver NPs being by far the most studied. Metal-based NPs present low toxicity to eukaryotic (mammalian cells) as they are able to differentiate prokaryotic (bacterial cells) from mammalian cells through bacteria’s metal transport system and metalloproteins [104]. When acting selectively with the bacterial cell, these NPs trigger antimicrobial action through three main routes: interaction with the lipid by-layer, interaction with cytosolic proteins and through oxidative stress due to the generation of reactive oxygen species (ROS) [104].

Giving their effectiveness as antibacterial agents, metal-based NPs have been used to develop antimicrobial packaging materials against a wide range of microorganisms in several food products (Table 6). Besides being used alone as active agents, metal-based NPs are also used in combination with bacteriocins, essential oils, and as a combination of several metal NPs [105,106,107]. However, the commercial use of packaging materials with NPs, from now on designated nanomaterials is still hindered by the lack of specific legislation and risk assessment data. In terms of regulation, the first requirement is to define what is a nanomaterial. From the EU point-of-view, the European Commission has recommended that a nanomaterial should contain a threshold of 50% of the particles in the number-based minimal external size distribution to be within the nanoscale (1–100 nm) [108]. However, this recommended definition is currently under review and has not been completely accepted. It is advised that all new nanomaterials follow the risk assessment procedure according to the guidelines provided by EFSA panels [108]. When working with materials containing nanoparticles, the first thing is to assess whether the nanoparticles can migrate from the packaging material into the food product. If no migration is observed or if the migration is within the desired limits, the safety assessment of the material should follow the regular directive for food contact materials [109]. If NP migration is observed, then, according to the EU Regulation on Novel Food (EU) No. 2015/2283, a food consisting of engineered nanomaterials will be considered a novel food and as such will require authorization [108]. To obtain such authorization and seal of EU approval, a complete risk assessment of the novel food shall be carried out by EFSA [110], which shall also be responsible for verifying that the most up-to-date test methods have been used to assess their safety [108].

#### 1.1.8. Phages

Bacteriophages have been acknowledged for their great effectiveness in controlling bacterial pathogens in agro-food industry [29]. Lytic phages are viruses able to infect and lyse bacterial cells and, as a consequence of microbial cell lysis, release large number of progeny phages, which can then continue the infection cascade [31]. As they are specific for a host cell, they do not interact with other microorganisms or eukaryotic cells in the environment and so, they do not cause illness neither in animals nor humans [31]. Besides this advantageous feature, phages are also easy and economically feasible to isolate and produce and, on opposite to other biological agents, they have a long shelf-life [29].

In terms of their application in food products for human consumption, in the last decade, FDA recognized some phage-based products as safe (GRAS) [86]. For instance, a commercial phage preparation called LISTEX™ P100 intended to be used on ready-to-eat meat and poultry products has already been approved for use as a food processing aid by Canada, United States of America and Netherlands [31]. Also, SalmoFresh™, a lytic *Salmonella* bacteriophage cocktail containing six Salmonella phages, has been granted GRAS status by the FDA [117]. Currently, also EFSA is evaluating the use of bacteriophages in food products with recommendations from EFSA’s BIOHAZ Panel that, to further assess phage safety in foods, it is necessary to evaluate the persistence of bacteriophages in foods and their ability to prevent recontamination with bacterial pathogens, research for specific combinations of bacteriophages, pathogens and foods [118].

Therefore, bacteriophages have great potential to be applied by the food industry as antimicrobial agents incorporated directly into food products or through their incorporation into the food packaging material for a more controlled release [30]. So far, packaging materials containing bacteriophages have proven to effectively control several foodborne pathogens, including *Salmonella enterica, Listeria monocytogenes* and *Escherichia coli* O157:H7 (Table 1). Given that phages are specific to a designated bacterial species, their use in food packaging usually depends on their target foodborne pathogen, meaning that the type of food product to be packaged is selected according to the main foodborne pathogen present in that food. Furthermore, nowadays, instead of incorporating one single phage in the packaging material, phage cocktails are preferred in order to broad the antimicrobial spectrum of the active material developed [31,119]. Also, when studying combinations of phages and other antimicrobials in order to increase phage antimicrobial activity, one has to consider that some chemical preservatives are capable of inactivating bacteriophages, meaning that combinations of bacteriophages and preservatives are less effective than either treatment alone (for a more detailed review please see [120]). In spite of their effectiveness as antimicrobials, the vast majority of these novel antimicrobial agents such as natural extracts, bacteriocins, essential oils and other present some challenges regarding their incorporation in the packaging films due to polymer-antimicrobial chemical incompatibilities, and also to the poor stability of some of the developed films due to antimicrobial instability issues [121]. So, over recent years, researchers have developed new formulations to alleviate these problems, mainly through the encapsulation of these antimicrobials. Besides protecting the antimicrobial compound, these encapsulation agents also yield a more controlled-release of the antimicrobial compound from the packaging material to the food product or packaging atmosphere. This is especially relevant in antimicrobial food packaging as a high concentration of released compound in the packaged food could result in sensory or legal issues, as compound concentrations can exceed the restriction limits; whereas low concentrations would not yield the antimicrobial efficiency needed and the new packaging would be useless [121].

## 2. Encapsulation Strategies for Antimicrobial Packaging

Encapsulation is defined as the process to entrap one substance (active agent) within another substance, yielding small particles that release their contents at controlled rates over prolonged periods of time and under specific conditions [122]. In the antimicrobial food packaging area, the encapsulation of antimicrobial compounds provides more efficient packaging materials by (i) protecting the antimicrobial compounds from degradation, volatilization or undesirable interactions with packaging materials, (ii) improving the compatibility between the packaging polymer and the antimicrobial substance, (iii) increasing the availability of the antimicrobial and (iv) providing a controlled release or/and stimuli-responsive release to extend the activity of the active material, reduce changes in food sensorial properties or comply with the legal restriction limits.

Encapsulating some types of antimicrobial substances has become essential to solve some problems that limit their use in packaging applications. In the case of EOs, for example, encapsulation is essential to reduce losses by volatilization or degradation during packaging manufacturing or storage, to improve the compatibility with biopolymer by increasing their solubility and/or to diminish the organoleptic impact in food products caused by their strong odour [123,124].

A broad range of delivery systems or carriers have been developed to encapsulate bioactive compounds in the food and pharmaceutical sectors such as cyclodextrins, liposomes, emulsions, nanoparticles or microcapsules [125]. However not all these available carriers can be applied in antimicrobial active packaging as they should be compatible with the packaging material and do not modify negatively their mechanical and physical properties in order to preserve their primary function of food protection. Herein, we review the most used systems for antimicrobial packaging development with emphasis in the novel strategies developed over the last five years.

### 2.1. Emulsions

**Figure 1:**
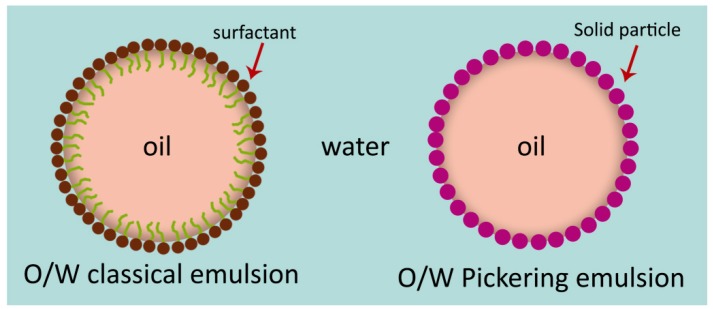
Schematic representation of a classical emulsion stabilized by surfactant and a Pickering emulsion stabilized by solid particles.

Conventional emulsions consist of two immiscible liquids where one liquid is dispersed in the other in form of small droplets (Figure 1). These colloidal systems can be used to encapsulate bioactive compounds at significant amounts. Lipophilic compounds can be encapsulated in oil-in-water (O/W) emulsions, while hydrophilic compounds can be encapsulated in water-in-oil (W/O) or oil-in-water emulsions. Multiple emulsions such as water-in-oil-in-water (W/O/W) and oil-in-water-in-oil (O/W/O) can also be used to encapsulate active compounds in order to improve delivery requirements [125,126].

Regarding antimicrobial packaging, emulsions are used almost exclusively to incorporate essential oils or their chemical constituents into water soluble polymers, generally of natural origin, resulting in an O/W emulsion. The incorporation of EOs in emulsions improves their compatibility with water-based polymers, provides more transparent films while protecting EOs from volatilization and enabling a more controlled released [127,128,129,130].

Emulsions with low particle size (nanometric or micrometric scale) present several advantages over systems containing larger particles [131,132] such as better stability, decreased particle aggregation, increased transparency, added rheological properties and higher bioavailability of the encapsulated substances. Therefore, presumably, antimicrobial films containing emulsions of low particle size will be more homogenous, transparent and effective than those prepared with conventional emulsions. In fact, this hypothesis has been demonstrated by several authors dealing with the encapsulation of EOs and their major components in water-based films. For example, Guo et al. demonstrated that films containing allyl isocyanate (AIT) microemulsions revealed stronger antimicrobial activity and were more homogenous than those containing conventional emulsions [133,134]. Similarly, Otoni et al. demonstrated that packaging films with nanoemulsions exhibited better uniformity and higher antifungal activity in packaged bread than those containing coarse emulsions [135]. Oh et al. found that chitosan edible films containing lemongrass oil nanoemulsions showed better antimicrobial activity and produced less sensorial changes in coated grape berries than similar coatings with higher droplet size [136].

Considering the advantages, most of the works carried out in recent years have focused their attention on the use of emulsion of lower particle size, namely microemulsions and nanoemulsions. Microemulsions are defined as oil and water colloidal dispersions stabilized by an interfacial layer of surfactant molecules with particles sizes ranging from 1 to 100 nm, usually 10–50 nm. This type of emulsions presents some advantages such as thermodynamic stability and transparency, which make them good vehicles to incorporate antimicrobial hydrophobic compounds into different polymeric matrices. However, they need a large amount of surfactant to be stable [137]. Nanoemulsions are defined as conventional emulsions containing very small particles, typically lower than 200 nm. Like conventional emulsions, they are thermodynamically unstable, but their lower droplet size endows them long-term stability, higher bioavailability and transparency. These nanoemulsions also required surfactants, but in a lower surfactant-to oil ratio than microemulsions. As disadvantages, they have low stability in acidic conditions and are usually prepared by high-energy methods such as high-pressure valve homogenization, ultrasonic homogenization or high-pressure microfluidic homogenization [137]. Nanoemulsions are, by far, the most used dispersions to encapsulate antimicrobials in active packaging. Examples of films containing micro and nanoemulsions recently developed together with their application to food systems are shown in Table 7.

Despite that, as can be seen, packaging materials containing emulsions as encapsulation strategy are based on polymers of natural origin. Most of the approaches used emulsifiers of synthetic origin, particularly, polysorbates such as Tween 20 [136,144,145] or Tween 80 [90,128,130,140,143,145,146,147,148,149,150]. Natural emulsifiers such as lecithin [97,138,141,151], soy protein isolate [141], arabinoxylan [133] or sapindus extract [152] have been scarcely used and generally in combination with polysorbates. Consequently, further research on the use of natural emulsifiers in bio-based packaging materials is on demand in order to satisfy the growing demand in food industry for natural ingredients.

Besides classical emulsions, Pickering emulsions have been used to encapsulate bioactive compounds with antimicrobial properties. These emulsions are stabilized by solid particles instead of the surfactants used in classical emulsions (Figure 1). As in the case of surfactants, stabilization of emulsion droplets takes place by adsorption of small solid particles at the surface of the emulsion droplets, although the mechanism of adsorption is very different to the one of surfactants [153]. This type of stabilization adds specific properties to Pickering emulsions which make them more suitable for certain applications. Particularly valuable for antimicrobial packaging applications is their higher stability and absence of surfactants [137,153]. Conversely, the main disadvantages of Pickering emulsions are their opacity and the limited number of stabilizing particles that can be used in food applications [137].

Additionally, it has been demonstrated that the use of this type of emulsions can improve some film characteristics when compared to those that incorporate classical emulsions. Almasi et al. compared pectin films incorporating oregano EO using nanoemulsions or Pickering emulsions [154]. The results showed that both have similar antimicrobial activity but the film containing Pickering emulsions present more suitable mechanical and water barrier properties. Moreover, oregano EO release is slower from films containing Pickering emulsions than from those containing nanoemulsions.

Despite the potential advantages of using Pickering emulsions, to date, few antimicrobial packaging materials have been developed using this technology (Table 8). Like in classical emulsions, Pickering emulsions are used as EO carriers and their components using solid stabilizing particles of natural origin. The antimicrobial activity of these new materials has been tested with good results in vitro, but only Fasihi et al. demonstrated their in vivo activity, namely, the inhibition of fungal growth in bread slices packaged in active films containing Pickering emulsions of rosemary essential oil [155].

### 2.2. Core-Shell Nanofibers: Emulsion and Coaxial Electrospinning

**Figure 2:**
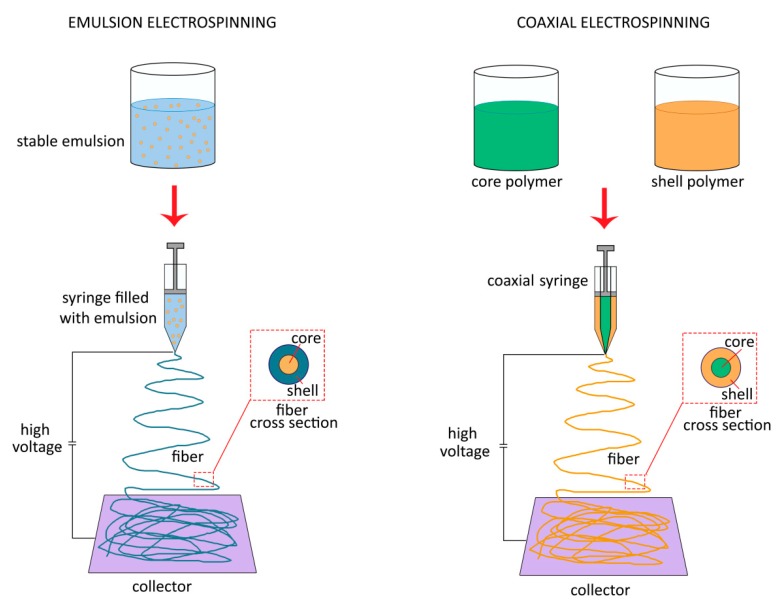
Emulsion electrospinning and coaxial electrospinning techniques.

Electrospinning is an effective, low cost and versatile technique used to produce continuous sub-micron or nano-scale fibrous films from various biopolymer materials such as chitosan, alginate, cellulose, dextran, gelatine or zein among others [158]. This technique is based in the use of high voltage electrostatic fields to charge the surface of a polymer solution droplet, thereby inducing the ejection of a liquid jet through a spinneret to form a nanofibrous film [158]. Electrospinning, particularly emulsion and coaxial electrospinning, can be used to produce nanofibers with core–shell morphology. Using this structure, bioactive compounds can be directly incorporated in the core protected by the shell layer minimizing their volatilization or oxidation and reducing their release ratio [159,160]. In emulsion electrospinning, a stabilized emulsion (W/O or O/W) can be used as spinning solution using the conventional electrospinning technology to obtain core-shell nanofibers (Figure 2). It has been shown that core-shell fibres produced by emulsion electrospinning are able to yield a more sustainable controlled released than fibres obtained by coaxial electrospinning despite the later having a more organized core-shell structure [161]. In coaxial electrospinning, two solutions (core and shell) are delivered separately through a coaxial capillary and drawn by electric field to generate nanofibers with core-shell morphology (Figure 2), meaning that this technique requires a more complex design than emulsion electrospinning and a precise control of different parameters such as interfacial tension and viscoelasticity of the two polymers [159,162].

Despite the attention drawn to electrospun core-shell nanofibers containing bioactive compounds in last years, the vast majority of research works are focused on pharmaceutical and biomedical fields while food applications have been less explored. However, the incorporation of antimicrobials in the core-shell nanofiber has shown a great potential to be used in active packaging materials, demonstrating a higher controlled-release and a strong antimicrobial action. Table 9 summarizes the developed antimicrobial packaging materials containing core-shell nanofiber as encapsulating strategy along with their antimicrobial activity and release performance data.

### 2.3. Cyclodextrins

**Figure 3:**
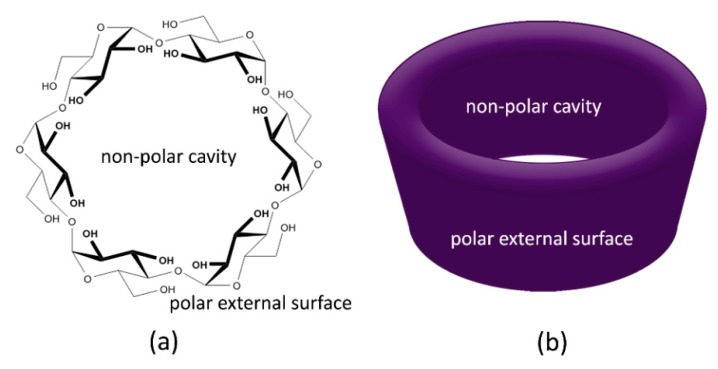
(**a**) Chemical structure and (**b**) geometrical shape of cyclodextrins.

Cyclodextrins (CDs) are a family of cyclic oligomers of α-d-glucopyranose linked by α-1,4 glycosidic bonds (Figure 3A) that can be produced due to the biotransformation of starch by certain bacteria such as *Bacillus macerans* [169]. The more common natural cyclodextrins are α- cyclodextrins (6 glucose subunits), β- cyclodextrins (7 glucose subunits) and ɣ- cyclodextrins (8 glucose subunits), being β-CD the cheapest and, therefore, the most used. CDs present a truncated conical cylinder shape with an inner non-polar cavity and a polar external surface that makes them capable to encapsulate hydrophobic substances (Figure 3B). The complex created between the CD and the loaded compound is called inclusion complex where CDs are the host molecules [169,170].

The use of CDs and modified CDs are one of the strategies most used in the food packaging area to encapsulate active compounds as indicated by the high amount of publications in the last fifteen years regarding this topic. Using this encapsulating strategy, the bioactive molecules improve their water solubility, can be protected from volatilization, oxidization or heating and can be released in a more controlled manner [171,172,173,174]. Moreover, the low price, semi-natural origin and non-toxic effects [169,170] of CDs explain the great interest of both research and industry in their use.

In last years, several of the publications dealing with cyclodextrins as encapsulation method in antimicrobial packaging have explored novel strategies to develop improved materials such as the incorporation of inclusion complexes in electrospun nanofibers.

As explained above, electrospinning is an effective and low cost technique to produce nanofibers mats. The fibrous film produced display high porosity, small pore size and high surface-to-volume ratio that make them more suitable to load high amounts of active substances [175]. The combined use of electrospun nanofibers with cyclodextrin inclusion complexes aim to combine the benefits provided by each technique at the same time. Wen et al. produced and tested polylactic acid film electrospun nanofibers containing cinnamon EO/β-CD inclusion complexes. The inclusion of cinnamon in the cyclodextrin improved its thermal stability and its antimicrobial action, probably due to a higher solubility. Moreover, the electrospun fibres containing the inclusion complex exhibited better antimicrobial activity and retain the EO better than those films prepared by casting [176].

Using the combination of both techniques, antimicrobial materials with improved properties have been developed. Recent research studies regarding the use of CD inclusion complexes in antimicrobial electrospun nanofibers are reviewed in Table 10.

Another recent strategy developed to encapsulate antimicrobial in CDs is the use of nanosponges [180]. Nanosponges are cross-linked cyclodextrin polymers nanostructured within a three-dimensional network that offer some advantages in respect to monomeric native cyclodextrins such as a higher loading capacity, increased protection of encapsulated compounds and better controlled released [181,182]. This novel approach has been used recently to encapsulate cinnamon and coriander essential oil demonstrating antimicrobial activity against foodborne Gram positive and Gram negative bacteria and a controlled EO release [181,182]. However, the incorporation of these novel structures in packaging materials has not been tested yet.

### 2.4. Halloysites Nanotubes

**Figure 4:**
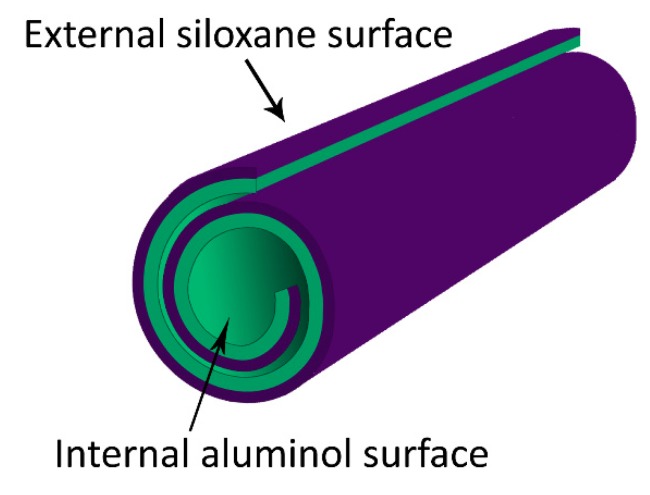
Halloysite nanotubes have an external surface composed of silanol (Si-OH) along with siloxane groups and an internal surface composed of aluminol (Al-OH) groups.

Halloysite nanotubes (HNTs) are a type of natural occurring aluminosilicate clay minerals which are available in abundance in many continents including Asia, North America, Europe, Oceania, and South America [183,184,185]. These substances display a characteristic two-layered (1:1) aluminosilicate structure similar to kaolin that usually adopt a hollow tubular nanostructure with a typical size of 500–1000 nm in length and 15–100 nm in inner diameter [186] (Figure 4). Owing to their tubular structure, HNTs can be used to load and release bioactive molecules, including antimicrobial agents. Furthermore, their low price, abundance, non-toxicity and eco-friendly features as well as their biocompatibility make them an attractive alternative to other tubular materials such as carbon nanotubes or TiO_2_ nanotubes [185,186].

Given the advantages described above, HNTs have been also applied in the antimicrobial packaging area. Several studies have demonstrated that the incorporation of antimicrobial substances via halloysite nanotubes improves the retention of the active compound in the packaging material and enables a more controlled-release. For example, a more extended lysozyme release from poly (ε-caprolactone) or poly(lactide) films has been achieved through its incorporation in HNTs [24,187]. Similarly, a slow release of rosmarinic acid from PLA films was obtained by including rosemary EO in halloysite nanotubes [188]. The use of HNTs to control the delivery rate has made it possible to increase the shelf-life of materials containing volatile antimicrobial agents. For example, films containing halloysite nanotubes loaded with thyme oil showed antimicrobial activity against *Escherichia coli* during 10 days after thymol was loaded into HNTs [189]. Similarly, LDPE lipid containing thymol/carvacrol/halloysite nanotubes retained their initial antimicrobial activity during 4 weeks of storage [190].

By being included in HNTs, antimicrobials can be protected from losses due to volatilization or other processes. For instance, in another study, carvacrol was encapsulated in halloysite nanotubes and subsequently incorporated into polyamide polymers by extrusion. The results showed that polymers containing halloysites retained approximately 90% of the initial carvacrol content; while for the control PA/carvacrol system, no residual carvacrol was detected due to carvacrol evaporation [191]. Similar results were obtained for LDPE containing halloysite nanotubes encapsulating mixtures of carvacrol and thymol [190].

Nonetheless, the incorporation of halloysites has also been related to negative effects as the incorporation of HNTs in starch films increased the opacity of the films and reduced the antimicrobial activity of the active starch [16].

Modifications in halloysites have been performed in order to obtain some advantages. For example, halloysites treated with NaOH have been used to increase the loading capacity of thyme oil from 180.73 to 256.36 (mg thyme oil/g HNT) [189]. Other studies demonstrated that the capping of HNTs both ends and/or the coating of the outer surface of the HNTs can be employed to modify the release rate of antimicrobial compounds. For instance, the capping of HNTs ends with sodium alginate and the coating of the surface with positively charged poly(ethylene imine) polymer using the layer-by-layer method, yielded a slower thyme EO release from HNTs [189]. Likewise, the coating of allyl isothiocyanate loaded HNTs with sodium polyacrylate (both ends and surface) enabled a more efficient release of AIT comparing to non-treated HNTs [192].

Halloysite-loaded film manufacturing has been made using different techniques that include classical methodologies as casting [16,187,188], compression moulding [187], extrusion [190] or more innovative ones such as electrospinning [24]. Besides, halloysites have also been incorporated in packaging materials as coatings [16,102,103] or inks [189].

The antimicrobials materials loaded with HNTs as carriers have demonstrated high in vitro antimicrobial activity [16,188,189,192,193]; notwithstanding, not all works carried out have applied this novel technology to food applications (Table 11)

### 2.5. Liposomes

**Figure 5:**
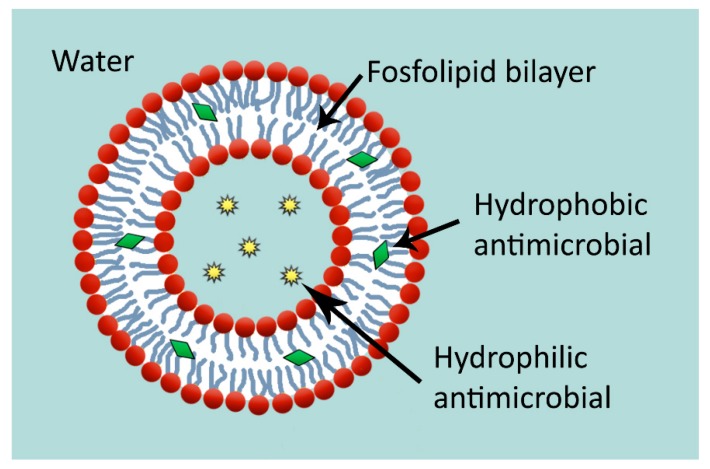
Liposome loaded with hydrophobic and hydrophilic antimicrobial substances.

Liposomes are microscopic spherical-shape vesicles composed of a wall of amphipathic lipids arranged in one or more concentric bilayers with a aqueous phase inside and between the lipid bilayers [196] (Figure 5). The ability of liposomes to encapsulate hydrophilic or lipophilic drugs have allowed these vesicles to become useful drug delivery systems, being one of most widely used carriers for antimicrobial agents [196]. Besides, the development of nanoliposomes has added the benefits of the nanosized particles to the encapsulation, delivery and targeting of bioactive compounds [197].

Using natural and non-toxic lipid molecules commercially available (generally lecithin and cholesterol), liposomes and nanoliposomes loaded with antimicrobial agents have been prepared and included in food packaging materials to obtain materials with improved properties. For example, the encapsulation of eugenol or cinnamon essential oils in lecithin liposomes led to chitosan films with higher retention ratio (40% − 50%) of volatile compounds when compared to what is retained when they are free incorporated by emulsification (1% − 2%) [198]. Moreover, the incorporation of cinnamon essential oil nanoliposomes in gelatine films allowed for a lower antimicrobial release rate together with an improvement of the antimicrobial stability [199]. Besides, coatings of chitosan loaded with *Satureja* plant essential oil nanoliposomes exhibited a prolonged and consistent antimicrobial activity on meat pieces during their storage time in comparison with coating containing free EO [200].

It is important to point out that liposomes can lead to negative changes in the optical properties of films due to the chromatic properties of lecithin or the occurrence of chemical reactions [198,201].

As can be seen in Table 12, several studies have tested films containing antimicrobial liposomes in food. For this purpose, natural extracts, EOs, phages, metallic nanoparticles or nisin have been included in liposomes and loaded in polymeric matrixes, especially in chitosan. Chitosan provides benefits from other biomaterials due to its intrinsic antimicrobial properties that can enhance the antimicrobial action of loaded liposomes.

Liposomes can also be further engineered to confer stimuli-responsive properties for drug delivery. Despite that these advanced structures have been widely applied in the biomedical area [207], only few developments have been carried out for food applications [177,208,209,210,211,212]. In the antimicrobial packaging field, only Lin et al. used this strategy to control the release of antimicrobials from the packaging material. In this work, cinnamon EO/β-cyclodextrin complexes were loaded into stimuli-responsive proteoliposomes, and subsequently incorporated in poly(ethylene oxide) electrospun nanofibers as strategy to control the growth of *Bacillus cereus* in beef. The mechanism of activation of these proteoliposomes is based in the degradation of casein present in liposome walls produced by *B. cereus* proteases [203].

### 2.6. Other Encapsulating Particles

Besides the previously mentioned encapsulation particles, other micro- or nanoparticles such as microcapsules, nanocapsules, nanostructured lipid carriers, solid-lipid nanoparticles or nanoparticles among others have been used to encapsulate flavours, vitamins, antioxidants, food colorants or antimicrobials for food applications [180,213]. However, not all these structures have been applied for antimicrobial encapsulation in active food packaging materials.

**Figure 6:**
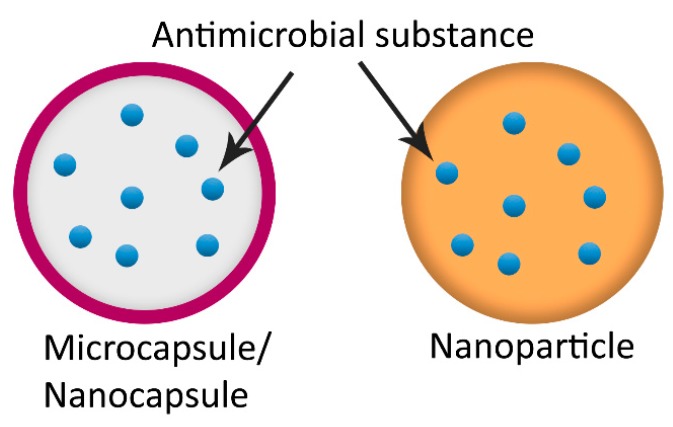
Microcapsule/nanocapsule and nanoparticle loaded with antimicrobial substances.

In the past years, responsive microcapsules and nanocapsules (Figure 6) containing antimicrobials agents have been incorporated in polymers to control the release, and consequently, improve its effectiveness. For instance, *Cymbopogon citratus* oil has been encapsulated in responsive microcapsules of gelatine-carboxymethylcellulose, gelatine-gum or melamine-formaldehyde walls. When these structures are subjected to mechanical stress, the wall breaks and the active compound is released. These responsive microcapsules have been incorporated in paper through coating, exhibiting antimicrobial activity against *Escherichia coli* and *Sacharomyces cerevisiae* in vapour phase after activation [214]. Similarly, thyme EO has been incorporated in responsive capsules of lightly cross-linked polyamide shell. The shell is responsive to light due to the trans–cis isomerization of the photochromic azo-moieties, which causes a contraction of the polymer chains leading the release of the encapsulated content [215]. These capsules have been incorporated in low-density polyethylene or poly(lactide) polymers by coating, releasing thyme EO with proved antimicrobial efficacy [216]. An innovative responsive microcapsule for the delivery of chlorine dioxide (ClO_2_) based on the reaction of NaClO_2_ with water and tartaric acid was developed by Huang et al. [34]. Poly (lactide) capsules were loaded with a gelatine core and NaClO_2_ and, afterwards, incorporated in PLA film containing tartaric acid. In the presence of water, ClO_2_ gas is produced and released from the film reducing the population of *Escherichia coli* and *Staphylococcus aureus* [34]. In a more recent work, this material was tested in vivo displaying a positive effect in food preservation by extending the shelf life of packaged mango [217].

Nanoparticles (Figure 6) have been also widely used in last years to encapsulate antimicrobials, generally EOs or their components, in diverse packaging materials. Antimicrobial-nanoparticle complexes of chitosan [218,219,220,221], silica [60,222], zein [223] and polylactide [224] have been incorporated into chitosan [218,219,224], gelatine [177,220] or cellulose [223], among others, with the attainment of antimicrobial activity both in vitro and in vivo. Food applications of recent works dealing with nanoparticles and microcapsules are listed in Table 13.

## 3. Conclusions and Future Trends

Due to green consumerism, plastic reduction and EU circular economy guidelines, the development of active antimicrobial packaging is currently transitioning from the use of non-biodegradable, non-compostable, oil-derived plastic materials with incorporated synthetic antimicrobial compounds such as organic acids and antibiotics, to the use of sustainable, environmentally-friendly, biodegradable packaging materials such as chitosan, zein, starch or cellulose, with incorporated natural-derived compounds such as essential oils, plant extracts and compounds, bacteriophages and bacteriocins, among others. This trend brought new challenges regarding the incorporation of these new products into these novel packaging materials in terms of antimicrobial-packaging material compatibilities and, the most important one, the decreased stability of these natural-based compounds as they are more prone to suffer degradation by heat and light. Furthermore, there are other losses to be faced when incorporating some of these antimicrobials in packaging materials due to their inherent volatilization, as in the case of essential oils and their major compounds. Consequently, the food industry and food scientists begin to search for new strategies that could alleviate these problems and, as a result, increase the durability and efficiency of these new natural-based antimicrobial packaging. At that moment, they turned to the novel strategies being tested for drug delivery as a possible answer for their problems. Biocompatible carriers such as cyclodextrins, liposomes, emulsions and halloysites have been explored as antimicrobial encapsulation systems for the development of new packaging materials. Notwithstanding, the industrial use of these encapsulation-based antimicrobial packaging materials is still hindered by several factors such as the cost of such vehicles, their EFSA approval status for the development of food contact materials and, also the costs associated with novel machinery and modifications in plants for the industrial production of these novel antimicrobial food packaging materials. Hence, to overcome these issues, better and cheaper encapsulating agents’ production methods are needed together with the investment in machinery scale-up so that the new antimicrobial materials manufactured can compete with the current ones, not only in terms of efficiency but also in terms of price.

## Figures and Tables

**Table 1:** Antimicrobial agents used in active food packaging. NA-not applicable.

Antimicrobial Class	Antimicrobial Agent	Packaging Material	Main Microorganisms	Food Product	Ref.
Organic acids	Lactic acid	Polyamide	*Escherichia coli* O157:H7	Fresh beef cuts	[10]
	Lactic acid	Chitosan pectin starch biocomposite	*Bacillus subtilis* *Listeria monocytogenes*	NA	[11]
	Sodium benzoate Citric acid	Polyvinyl alcohol (PVA)	*Staphylococcus aureus* *Escherichia coli* *Candida albicans*	NA	[12]
	Potassium sorbate	Fish collagen and polyvinyl alcohol (PVA) composite	*Escherichia coli* *Staphylococcus aureus*	NA	[13]
Bacteriocins	Sakacin-A	PE coated paper	*Listeria monocytogenes*	Thin-cut meat	[14]
	Sakacin-A	Cellulose nanofibres	*Listeria monocytogenes*	Smoked salmon fillets	[15]
	Nisin	Starch-halloysite nanocomposites	*Listeria monocytogenes* *Clostridium perfringens*	NA	[16]
	Pediocin	Starch-halloysite nanocomposites	*Listeria monocytogenes* *Clostridium perfringens*	NA	[16]
	Nisin	Chitosan-carboxymethylchitosan composite films	*Listeria monocytogenes*	NA	[17]
	Bacteriocin 7293	Poly (lactic acid)/sawdust particle biocomposite film	*Listeria monocytogenes* *Staphylococcus aureus* *Pseudomonas aeruginosa* *Aeromonas hydrophila* *Escherichia coli* *Salmonella Typhimurium*	Pangasius fish fillets	[18]
	Bacteriocin-like substances	Starch	*Listeria monocytogenes*	Cheese	[19]
	Bacteriocin-like substances	Triticale flour films	*Listeria innocua*	Cheese	[20]
	Bacteriocin-producer living bacteria	Poly (ethylene terephthalate) (PET) coated with polyvinyl alcohol (PVOH)	*Listeria monocytogenes*	Precooked chicken fillets	[21]
Enzymes	Lysozyme	Nonwoven regenerated cellulose with carbon nanotubes and graphene oxide	*Micrococcus lysodeikticus*	NA	[22]
	Lysozyme+ lactoferrin	Carboxymethyl cellulose-coated paper	*Listeria innocua* *Escherichia coli*	Veal carpaccio	[23]
	Lysozyme	Polyamide 11 (PA11) with halloysite nanotubes (HNTs)	Pseudomonads	Chicken slices	[24]
	Glucose oxidase	Whey protein isolate	*Listeria innocua* *Brochothrix thermosphacta* *Escherichia coli* *Enterococcus faecalis*	NA	[25]
	Lactoperoxidase	Chitosan	*Shewanella putrefaciens* *Pseudomonas fluorescens* Psychrotrophs Mesophiles	Rainbow trout	[26]
Biopolymers	Chitosan	Chitosan/ethylene copolymer	*Escherichia coli* *Salmonella* Enteritidis *Listeria monocytogenes*	NA	[27]
	Hydroxyethyl cellulose/sodium alginate	NA	*Escherichia coli* *Staphylococcus aureus*	NA	[28]
Bacteriophages	ϕIBB-PF7A	Alginate	*Pseudomonas fluorescens*	Chicken fillets	[29]
	vB_EcoMH2W	Chitosan	*Escherichia coli* O157:H7	Tomatoes	[30]
	LISTEX™ P100	Cellulose membranes	*Listeria monocytogenes*	Ready-to-eat turkey	[31]
Other	LAE	Cellulose nanofibres	*Listeria monocytogenes*	NA	[32]
	Sulphur nanoparticles	Chitosan	*Listeria monocytogenes* *Escherichia coli*	NA	[33]
	Chlorine dioxide	PLA	*Staphylococcus aureus* *Escherichia coli*	NA	[34]
	Quaternary ammonium salt	PVA/starch	*Staphylococcus aureus* *Bacillus subtilis* *Escherichia coli* *Pseudomonas aeruginosa*	NA	[35]

**Table 2:** Organic acids or organic acid-derived compounds listed as food preservatives and their E-numbers.

Compound	E Number
Sorbic acid	E200
Potassium sorbate	E202
Calcium sorbate	E203
Benzoic acid	E210
Sodium benzoate	E211
Potassium benzoate	E212
Calcium benzoate	E213
Ethyl *p*-hydroxybenzoate	E214
Sodium ethyl *p*-hydroxybenzoate	E215
Methyl *p*-hydroxybenzoate	E218
Sodium methyl *p*-hydroxybenzoate	E219
Acetic acid	E260
Potassium acetate	E261
Sodium acetate	E262
Calcium acetate	E263
Lactic acid	E270
Propionic acid	E280
Sodium propionate	E281
Calcium propionate	E282
Potassium propionate	E283

**Table 3:** Examples of bacteriocins used in food packaging.

Bacteriocin	Characteristics	Producer	Target Microorganisms	Ref.
Nisin	Heat stable at 121 °C (pH = 2) Less stable at pH 5–7	*Lactobacillus lactis* subsp. *lactis*	*Streptococcus thermophilus* *Lactobacillus spp.* *Listeria monocytogenes* *Lactobacillus lactis* *Staphylococcus aureus* *Clostridium botulinum* *Bacillus cereus*	[43]
Lacticin 3147A	Heat stable at 100 °C (10 min at pH 5) Stable at room and low temperature Most stable at acid and neutral pH	*Lactobacillus lactis* DPC3147	*Bacillus subtilis* *Staphylococcus aureus* *Listeria monocytogenes* *Lactobacillus fermentum*	[44]
Pediocin PA-1	Stable at pH 4 to 6, becomes less stable as pH increases. Heat stable at 80 °C (10 min)	*Pediococcus acidilactici*	*Lactobacillus helveticus* *Pediococcus pentosaceus* *Listeria monocytogenes*	[43]
Enterocin AS-48	Remarkably stable to extremes of pH and denaturing agents Inactivated by heat at 65 °C and alkaline pH Compatible with several chemical compounds such as EDTA, lactic acid and sodium hypochlorite	*Enterococcus faecalis* subsp. *liquefaciens* S-48	*Corynebacterium spp.* *Mycobacterium spp.* *Nocardia spp.* *Micrococcus spp.* *Staphylococcus spp.* *Listeria monocytogenes* *Brochothrix thermosphacta* Lactic acid bacteria *Bacillus cereus* *Bacillus coagulans* *Bacillus subtilis* *Clostridium perfringens* *Clostridium sporogenes* *Clostridium tetani* *Myxococcus spp.* *Escherichia coli* *Rhizobium spp.* *Agrobacterium spp.* *Salmonella spp.* *Shigella spp.* *Pseudomonas spp.* *Klebsiella spp.*	[45]
Sakacin-A	Heat-stable (100 °C, 20 min) Active at pH 2–9 Most stable at pH 3–5 Stable during frozen storage	*Lactobacillus sakei* Lb706	*Listeria monocytogenes* *Listeria innocua* Lactic acid bacteria	[43,46]
Bacteriocin 7293	Stable in organic solvents and high ranges of pH and temperature	*Weisella hellenica* BCC 7293	*Pseudomonas aeruginosa* *Aeromonas hydrophila* *Salmonella* Typhimurium *Escherichia coli*	[42]

**Table 4:** Natural extracts and compounds (with the exception of essential oils and their components) used for the development of active food packaging. NA-not applicable.

Natural Compound	Packaging Material	Antimicrobial Activity	Food Preservation Data	Ref.
Gallic acid	Chitosan coating	Total viable counts	The addition of 0.2% gallic acid to chitosan films for pork loin coating showed antioxidant and antimicrobial properties under high oxygen MAP storage at 4 °C	[61]
Lignign	Hydroxypropylmethylcellulose composite	*Brochotrix thermosphacta* *Pseudomonas fluorescens*	NA	[67]
Curcumin	Chitosan	*Staphylococcus aureus* *Escherichia coli*	NA	[60]
Pinosylvin	Cellulose/polypropylene absorbent pads	*Campylobacter jejuni* *Campylobacter coli* Total viable counts Pseudomonads Psychrotrophs Lactic acid bacteria	At 4 °C, pads with 0.4 mg pinosylvin/cm2 exhibited anti-*Campylobacter* activity in chicken fillets and exudates Active coated pads were not able to reduce pseudomonads but caused reductions in lactic acid bacteria, psychrotrophs and total viable counts	[68]
Resveratrol	Polyethylene (PE) film polypropylene (PP) film	*Staphylococcus aureus* *Escherichia coli*	NA	[69]
Murta fruit extract	Methyl cellulose films	*Listeria innocua*	NA	[70]
Green tea extract	Chitosan	Total viable counts, Yeasts Moulds Lactic acid bacteria	Decreased number of total viable counts, lactic acid bacteria, yeasts and moulds in film-wrapped pork sausages stored at 4 °C for 20 days	[71]
*Allium ursinum* L. extract	Poly(lactic acid) (PLA) film	*Staphylococcus aureus* *Escherichia coli*	NA	[72]
*Ginkgo biloba* extract	Gelatine film	*Staphylococcus aureus* *Candida albicans*	NA	[73]
Spirulina extract	Chitosan film	*Escherichia coli* *Staphylococcus aureus* *Pseudomonas aeruginosa* *Listeria monocytogenes* *Salmonella typhimurium* *Bacillus subtilis* *Bacillus cereus*	NA	[74]
Turmeric extract	Chitosan film	*Staphylococcus aureus* *Salmonella spp.*	NA	[75]
Grapefruit seed extract	Poly(lactide)/poly(butylene adipate-co- terephthalate) composite film	*Listeria monocytogenes*	NA	[76]
Olive leaf powder and extract	Gelatine	*Listeria monocytogenes*	Films with 5.63% (w/w) of olive leaf extract decreased *L. monocytogenes* growth rate on inoculated RTE cold-cold-smoked salmon	[77]
Citrus extract	Chitosan	*Listeria innocua*	NA	[78]
Kombucha tea extract	Chitosan	*Staphylococcus aureus* *Escherichia coli* Total viable counts *Staphylococcus spp.*	Decrease in total viable and staphylococci counts in minced beef packaged with active films at 4 °C The shelf life of stored minced beef packaged in chitosan/kombucha tea can be extended up to 6 days	[79]
Propolis extract	Chitosan/cellulose nanoparticles film	Total viable count Psychrotrophic bacteria *Pseudomonas spp.* Lactic acid bacteria Enterobacteriaceae	Films containing propolis extract 2% and cellulose nanoparticles delayed microbial growth as well as lipid and protein oxidation of minced beef meat	[80]

**Table 5:** Essential oils and their components and their use for the development of active food packaging.

Essential oil Component	Encapsulation Strategy	Packaging Material	Food Product	Antimicrobial Effectiveness in vivo	Ref.
Cinnamon	NA	Polyvinyl alcohol electrospun fibres	Strawberries	When compared to control films, EO films stopped fungal rotting for up to 6 days of storage at 21 °C	[89]
Oregano	Nanoemulsion	Mandarin fibre edible coating	Low-fat cut cheese	Decreased *Staphylococcus* microbial population by 1.4 and 1.5 log CFU/g in coated cheese pieces containing 2.0% or 2.5% w/w of EO, respectively, during 15 days of refrigerated storage	[90]
Lemongrass	NA	Zein edible coating	Cold-smoked sunshine bass fillets	LG-treated samples reduced *L. monocytogenes* counts by 2.5 log in polyvinyl chlorine and 1.7 log in vacuum-packaged samples, respectively	[91]
Ginger	NA	Soy protein/zein electrospun fibres	Fresh Minas cheese	Significant reductions of *L. monocytogenes* were observed on the 3^rd^ and 9^th^ day of storage At day 9, *L. monocytogenes* counts decreased from 4.39 log CFU/g to 3.62 log CFU/g for the stored cheeses in the package containing EO-fibres when compared to the cheese stored in the fiberless package at 4 °C	[92]
Thymol Carvacarol	Montmorillonite	Themoplastic starch films	Strawberries	*In vivo* additive/synergistic antimicrobial effect over *Botrytis cinerea*-inoculated strawberries was observed when carvacrol+thymol were both included in the films with respect to the films containing only carvacrol A drastic reduction of 2.4-fold on EO inhibitory concentration against *Botrytis cinerea* in strawberries stored at room temperature for 5 days: IC_50_ values dropped from 14.16 g/kg film (only carvacrol) to 5.90 g/kg film (carvacrol: thymol 50:50) in indirect contact with the films	[93]
*Paulownia tomentosa*	Chitosan nanoparticles	Chitosan edible coating	Pork chop slices	EO-chitosan coatings decreased microbial growth (total viable counts, Pseudomonads and lactic acid bacteria) on pork chops compared to the control during 16 days of refrigerated storage Microbial shelf-life extension from 6 to 9 days	[94]
Thyme Cinnamon Lemongrass	NA	Chitosan film	Peanut kernels	Peanut kernels packed in chitosan films incorporated with 4% cinnamon EO showed complete inhibition of *Aspergillus flavus* and *Penicillium citrinum* growth at 4 and 28 °C after 24 days of storage compared with all other treatments Thyme and lemongrass EOs were less effective in reducing fungal growth at all concentrations and conditions tested	[95]
Chamomile Ginger	NA	Whey protein isolate edible coating	Rainbow trout fillets	Significant reduction in total viable counts and psychrotrophs was observed in trout fillets during 15 days of refrigerated storage when coated with ginger and camomile alone or in combination The best results were obtained when both oils were used in combination These films did not show a significant reduction in lactic acid bacteria counts and Pseudomonads	[96]
Oregano Tea tree Peppermint	Nanoemulsion	Cellulose nanocrystals (CNCs) reinforced chitosan	Rice	Of the 3 combinations tested (thyme:oregano, thyme:tea tree and thyme:peppermint), thyme:oregano nanoemulsions were the most effective against *A. niger, A. flavus, A. parasiticus* and *P. chrysogenum* Thyme:oregano films caused a significant reduction in all moulds growth during the 8 weeks of storage at room temperature This antifungal activity was improved when active films were used in combination with irradiation treatment	[97]
Geraniol α-Terpilenol	NA	Ethylene–vinyl alcohol copolymer (EVOH)	Fish slices	On day 8 of 10 days of refrigerated storage, the total viable counts cut down 1.98 ± 0.02 log units for fish samples packaged in geraniol/EVOH films Active films containing 6% (w/w) of geraniol and terpineol effectively extended shelf life by 4–5 days under cold-storage conditions compared with the control group	[98]
Eugenol Carvacrol Thymol	NA	Zein edible coating	Melons	The coating of melons with zein-2% eugenol mixtures caused a marked and similar decrease in both *L. innocua* and *E. coli* counts on melon surface during storage at 4 °C for 10 days	[99]
Cumin	NA	PET films coated with chitosan and alginate	Chicken meat	No significant growth reduction was obtained for total viable counts and psychrotrophs in active film chicken samples during refrigerated storage during 6 days	[100]
Thyme	NA	Silk fibroin electrospun fibres	Poultry (chicken and duck) meat	Active films caused a 2-fold reduction on *Salmonella* Typhimurium on chicken and duck meat stored at 25 and 4 °C Films antimicrobial activity was enhanced when combined with cold plasma	[101]
EO mix (carvacrol:oregano:cinnamon 70:10:20)	Cyclodextrin Halloysite tubes	Cardboard	Tomatoes	Decay incidence of tomatoes within cyclodextrin−EOs boxes was reduced from 9−15% to 2% after a storage period of 6 days/8 °C+12 days/25 °C	[102]
Carvacrol	Halloysite tubes	Chitosan-coated polyethylene	Chicken meat	Active films caused a 1.5 log reduction on total viable counts on chicken meat surface following 24h of incubation at 4 °C	[103]

**Table 6:** The use of metal nanoparticles in antimicrobial food packaging.

Metal NP.	Packaging Material	Food Product	Antimicrobial Effectiveness	Ref.
Bimetallic silver–copper (Ag–Cu)	Polylactide (PLA) + cinnamon EO films	Chicken meat	PLA films with 4% of bimetallic NPs reduced *L. monocytogenes, S. typhimurium* counts by 1 log CFU/g and *C. jejuni* counts by 3 log CFU/g during refrigerated storage for 150 days	[107]
Zinc oxide (ZnO)	Starch films	Fresh-cut mushrooms	Films with 3% ZnO exhibited antimicrobial activity against *L. monocytogenes*, resulting in a reduction of 0.86 log CFU/g after 6 days of storage at 4 °C in polypropylene containers	[111]
Titanium oxide (TiO_2_)	Low-density polyethylene (LDPE)	Fresh minced meat	ZnO nanoparticle (2%)-coated LDPE films were identified as the best case to improve shelf life and prevent *E. coli* growth in fresh calf minced meat during refrigerated storage for 72 h	[112]
Silver	Polyvinyl alcohol-montmorillonite blend	Chicken sausages	Marked reduction (qualitative) of total viable cell counts in chicken sausage samples stored at 4 °C for 4 days	[113]
Silver	Polyethylene (PE) + clay blend	Chicken breast	Films containing 5% Ag and 5% TiO_2_ had the greatest effect on decreasing the microbial load of the chicken sample contaminated with *S. aureus* for 5 days at 4 °C Films were more effective in inhibiting the growth of *S. aureus* than *E. coli*	[114]
Zinc oxide	Polylactide/poly(ε-caprolactone) + clove EO	Scrambled eggs	The efficacy of the composite films was verified against *S. aureus* and *E. coli* inoculated in scrambled egg, and results indicated that the PLA/PEG/PCL/ZnO/CEO film exhibited the highest antibacterial activity during 21 days storage at 4 °C	[115]
Zinc oxide	Gelatin-chitosan nanofibers composite film	Chicken Cheese	The results showed that the wrapping with nanocomposite film significantly decreased the growth of inoculation bacteria in chicken fillet and cheese samples stored at 4 °C for 12 days *S. aureus* and *E. coli* cell counts (chicken) were reduced by 2 log CFU/g during storage, whereas in cheese samples, *P. aeruginosa* and *E. coli* were reduced by only 1 log CFU/g	[116]

**Table 7:** Antimicrobial packaging materials loaded with antimicrobial nanoemulsions or microemulsions and their application in food.

Packaging Material	Encapsulated Antimicrobial	Surfactant	Food Application	Antimicrobial Activity	Ref.
Carboxymethyl chitosan film	Carvacrol	fatty alcohol polyoxyethylene ether carboxylic acid	Wheat bread exposed to active films without direct contact	Reduction of aerobic mesophilic bacteria, mould and yeast growth	[138]
Chitosan film or edible coating	Allyl isothiocyanate (AIT) or lauric arginate ester (LAE)	Corn-bio-fibre gum	Packaged ready to eat deli turkey	Inhibition of inoculated *Listeria innocua* growth by AIT or LAE	[134]
			Coated strawberries	Reduction of the survival of inoculated *Escherichia coli* O157:H7 and *Salmonella spp*., especially with AIT films	
Quinoa/chitosan edible coating	Thymol	Tween 80/Miglyol 812	Coated strawberries	Reduction of yeast and fungal growth	[139]
Sodium caseinate edible coating	Ginger EO	Tween 80	Coated chicken fillets	Reduction of aerobic psychrophilic bacteria, moulds and yeasts growth	[140]
Reinforced chitosan films with cellulose nanocrystals	Thyme-oregano EO mixture	Lecithin and Tween 80	Packaged rice	Inhibition of fungal growth The inhibitory effect was increased when gamma irradiation was also applied	[97]
Soybean polysaccharide edible coating	Cinnamon EO	Soy protein isolate and lecithin	Coated beef meat	Reduction of aerobic psychrophilic bacteria, moulds and yeasts growth	[141]
Jujube gum (JG) edible coating	Nettle EO	Tween 40	Coated beluga sturgeon fillets	Reduction in total and psychrotrophic bacterial counts	[142]
Sodium alginate and mandarin fibre edible coating	Oregano EO	Tween 80	Coated low-fat cheese pieces	Reduction of psychrophilic bacteria growth and inhibition of mould and yeast growth Lower *Staphylococcus aureus* survival in inoculated cheese	[90]
Pectin edible coating	Cinnamon bark and garlic EOs and curcumin	Tween 80	Coated breast chicken fillet	Reduction of total and psychrophilic bacteria, yeast and mould growth	[143]

**Table 8:** Pickering emulsions used in antimicrobial packaging as encapsulating strategy.

Emulsified Antimicrobial	Stabilizing Solid Particles	Antimicrobial Activity	Ref.
Rosemary EO	Carboxymethyl cellulose/polyvinyl alcohol	*In vitro* antifungal activity against *Penicillium digitatum* Inhibition of fungal growth in packaged bread slices	[155]
Thymol	Zein/chitosan complex particles	Slight in vitro antimicrobial activity against *Escherichia coli* and *Staphylococcus aureus*	[156]
Marjoran EO	Whey protein isolate/inulin	*In vitro* antimicrobial activity against *Escherichia coli* and *Staphylococcus aureus*	[154]
Oregano EO	Soluble soybean polysaccharide/soluble soy protein	*In vitro* antimicrobial activity against *Escherichia coli* O157:H7, *Pseudomonas aeruginosa* and *Staphylococcus aureus*	[157]

**Table 9:** Electrospun core-shell nanofibers loaded with antimicrobial compounds.

Antimicrobial	Nanofiber Material	Technique of Fabrication	Antimicrobial Release Performance	Antimicrobial Action	Ref.
Orange EO	Zein prolamine	Coaxial electrospinning	Higher retention of EO in the film as increasing the amount of zein prolamine	Antimicrobial activity in vitro against *Escherichia coli*	[163]
Curcumin	poly(vinyl alcohol) - chitosan	Coaxial electrospinning	Extended release of curcumin from the material	Inhibition of methicillin-resistant *Staphylococcus aureus* and *Staphylococcus epidermidis* growth *in vitro*	[164]
Phytoncide	poly(vinyl alcohol)	Emulsion electrospinning	Sustained release of phytoncide from the film over 14 days	Reduction of *Staphylococcus aureus* and *Escherichia coli in vitro*	[165]
Cinnamon	Polyvinylpyrrolidone	Emulsion electrospinning	ND	Antibacterial activity against *Staphylococcus aureus*, *Escherichia coli*, and *Candida albicans in vitro*	[166]
Thymol	Poly(lactide-co-glycolide)	Coaxial electrospinning	ND	Reduction of microbial growth and increase the shelf life of strawberries packaged in the active material	[167]
Eugenol	Polyvinyl pyrrolidone (core) and shellac (shell)	Coaxial electrospinning	Slower release of thymol from the film	Extension of shelf life of strawberries packaged with the active fibrous film	[168]

**Table 10:** Electrospun nanofibers loaded with antimicrobial inclusion complexes.

Nanofiber Material	Inclusion Complex	Antimicrobial Activity	Reference
Gelatine	Thyme EO/β-cyclodextrin ε-polylysine	Reduction in bacterial counts in coated chicken meat without adverse impact on colour, texture and sensory evaluation	[177]
Polyvinyl alcohol	Cinnamon EO/β-cyclodextrin	Higher in vitro antibacterial against *Staphylococcus aureus* and *Escherichia coli* than nanofibers without cyclodextrins Reduction of bacterial counts and increasing of shelf life of wrapped mushrooms	[178]
Poly(ethylene oxide)	Tea tree oil/β-cyclodextrin	Antibacterial activity against *Escherichia coli* O157:H7 After plasma treatment the film show enhanced antibacterial activity due a higher release rate	[177]
Zein	Eucalyptus EO/β-cyclodextrin	*In vitro* antimicrobial activity *Staphylococcus aureus* and *Listeria monocytogenes*	[179]
Zein	Thymol/γ-Cyclodextrin	Higher antimicrobial activity *Escherichia coli* and *Staphylococcus aureus* in vitro than nanofibers with non-encapsulated thymol Reduction of bacterial count in meat stored up to 5 days at 4 °C	[171]
Polylactic acid	Cinnamon EO/β-cyclodextrin	Antimicrobial activity against *Escherichia coli* and *Staphylococcus aureus* in vitro and efficacy in reducing bacterial counts in packaged pork film	[176]
Polyvinyl alcohol	Cinnamon EO/β-cyclodextrin	Antimicrobial activity against *Escherichia coli* and *Staphylococcus aureus* in vitro and extension of the shelf life of packaged strawberries	[89]

**Table 11:** Application of antimicrobial packaging materials containing loaded halloysites nanotubes to food applications.

Encapsulated Antimicrobial	Packaging Material	Food Application	Results	Ref.
Carvacrol	Polyamide film	Cherry tomatoes, lychee and grapes packaged in bags	Decay reduction in all foods except in cherry tomatoes packed in high concentrations of carvacrol	[191]
Carvacrol	Low density polyethylene film	Inoculated sliced wheat bread exposed to active films in a vapour phase assay	Inhibition of fungal growth after 11 days at 25 °C Films containing encapsulated carvacrol showed better performance than those without encapsulation	[194]
Lysozyme	Polyamide nanofibers	Chicken slices were stored on pads of active nanofibers	Reduction of *Pseudomonas* growth by 1-2 log CFU/g during storage at 4 °C	[24]
carvacrol	Polyethylene coated with chitosan loaded with HNTs	Wrapped chicken meat	Reduction of bacterial counts in 1.4 log CFU/cm^2^ (1 log more than films without HNTs)	[103]
Carvacrol and thymol mixtures	Low density polyethylene film	Inoculated and diluted hummus exposed to active films in a headspace assay	Inhibition of *Escherichia coli* growth after 22 h at 27 °C	[190]
Carvacrol	Low density polyethylene/poly ethylene vinyl alcohol layered films	Inoculated cherry tomato exposed to active films using a packaged simulation	Inhibition of *Alternaria alternata* and *Rhizopus spp*. growth after 4 days at 23 °C	[195]
Carvacrol, oregano and cinnamon EOs mixture	cardboard box coated with a lacquer loaded with HNTs	Fresh tomatoes stored in active cardboard boxes	Some microbial reduction after 6 days of storage	[102]

**Table 12:** Use of antimicrobial materials containing antimicrobial liposomes in food applications.

Antimicrobial	Liposomes	Packaging Material	Food Application	Antimicrobial Activity	Reference
*Escherichia coli* 0157:H7 phage	Lecithin and cholesterol	Chitosan film	Wrapped beef	Extended antibacterial activity against *Escherichia coli* O157:H7 in inoculated beef Extension of beef shelf life without affect sensorial properties	[201]
*Artemisia annua* oil	Lecithin and cholesterol	Chitosan edible film	Coated cherry tomatoes	*Escherichia coli* 0157:H7 growth reduction without changes in overall like mouthfeel and texture Changes in the colour were observed	[202]
Cinnamon EO	Lecithin, cholesterol and casein	Poly(ethylene oxide) nanofibers	Packaged beef	Reduction of inoculated *Bacillus cereus* with no impact on sensorial properties	[203]
Nisin or nisin-silica	Lecithin and cholesterol	Chitosan edible coating	Coated cheese	Extended antibacterial activity against inoculated *Listeria monocytogenes* maintaining the sensory properties of cheese	[204]
Cinnamon	Lecithin	PVA electrospun nonwoven	Packaged shrimp	Antibacterial activity against total bacteria and *Pseudomonas aeruginosa*	[205]
Laurel EO and lignin-silver nanoparticles	Phosphatidyl choline and choresterol	Chitosan coated in polyethylene films	Packaged pork meat	Increase of pork meat self-life by reduction of TVB-N values and keeping the quality of pork	[206]

**Table 13:** Antimicrobial materials containing active nanoparticles or microcapsules applied in food.

Particle Matrix	Encapsulated Antimicrobial	Antimicrobial Packaging	Food Application	Results	Reference
Chitosan microcapsules	Sorbic acid	Ethylene vinyl alcohol copolymer/polyethylene terephthalate film	Packaged snakehead	Increased the shelf life 4 days by reducing total volatile counts	[225]
Chitosan microcapsules	Grape seed extract and carvacrol	Chitosan films	Packaged salmon	Lower total volatile basic nitrogen and bacterial counts for a longer period of time.	[226]
Chitosan microcapsules	Cinnamon EO	Layer by layer edible coating of alginate and chitosan loaded with cinnamon microcapsules	Coated mangoes	Extension of mango shelf life. Reduction of black spots produced by moulds	[227]
Poly-γ-glutamic acid/chitosan nanoparticles	Nisin	Polyethylene oxide nanofibers coated in aluminium foil	Packaged cheese	Antibacterial activity against *Listeria monocytogenes* on cheese, without impact on the sensory quality	[228]
Chitosan nanoparticles	Clove oil	Krafted Gelatine nanofibers coating	Packaged cucumber	Inhibition of *Escherichia coli* O157:H7 biofilms in cucumber	[220]
Zein nanoparticles	Silymarin	Bacterial cellulose nanofiber films	Packaged salmon	Reduction of total volatile basic nitrogen contents during storage	[223]
Chitosan nanoparticles	Moringa oil	Gelatin nanofibers	Packaged cheese	Antibacterial effect against *Listeria monocytogenes* and *Staphylococcus aureus* on cheese at 4 °C and 25 °C without impact on the surface colour and sensory quality	[221]
